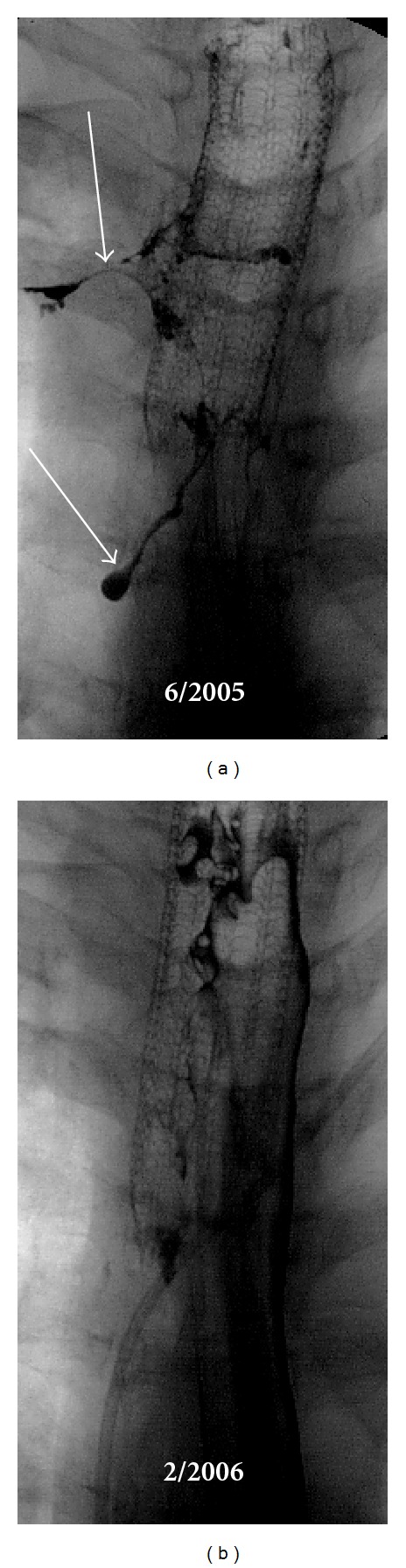# Hodgkin Lymphoma Untreated for Six Years Presenting with Tracheoesophageal Fistula

**DOI:** 10.1155/2012/457908

**Published:** 2012-07-02

**Authors:** Jason R. Westin, Amye Gibbs, Keith R. Mills, Sattva S. Neelapu

**Affiliations:** ^1^Department of Lymphoma and Myeloma, Division of Cancer Medicine, The University of Texas MD Anderson Cancer Center, Houston, TX 77030, USA; ^2^Campbell County Memorial Hospital, Gillette, WY 82717, USA

## Abstract

Hodgkin lymphoma is a highly curable cancer with modern therapy, with five-year survival rates in excess of 80%. However, the natural history of the untreated disease is largely unknown. We present the case of a patient with Hodgkin lymphoma who went untreated for over 5 years due to patient choice. Eventually, the patient developed hypoxemia, significant weight loss, and a tracheoesophageal fistula. After a placement of a gastrostomy tube and tracheal stent, treatment with standard chemotherapy was initiated. The patient achieved an excellent response, the fistula closed without further intervention, and there is no evidence of relapse six years later. Even in a patient with significant Hodgkin-lymphoma-related sequelae, standard therapy can result in excellent long-term outcomes.

## 1. Introduction

There are over 60,000 new diagnoses of Hodgkin lymphoma worldwide each year, and the vast majority are expected to achieve long-term survival with modern therapy [1, 2]. As Hodgkin lymphoma is now effectively curable in most patients, it is no longer considered a “dreaded disease”—however, this was not always the case. Prior to the development of combination chemotherapy, Hodgkin lymphoma was treated with surgery, radiation therapy, and/or single agent chemotherapy with poor outcomes. We present the highly unusual, yet instructive, case of a patient with biopsy-proven Hodgkin lymphoma who refused conventional therapy for 5 years, eventually consenting to chemotherapy after the development of morbid complications. 

## 2. Case Presentation

**Figure 1:**
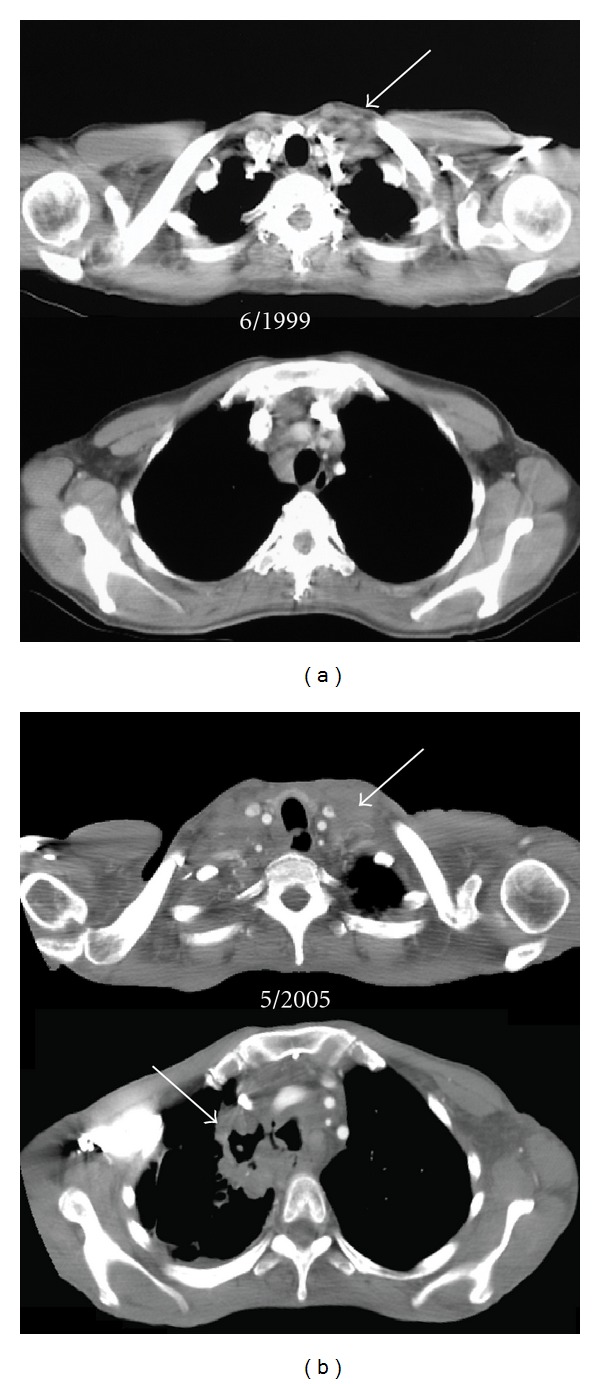


A 53-year-old woman without significant past medical history developed an asymptomatic left-sided supraclavicular mass. A core needle biopsy showed sheets of large neoplastic cells that were positive for CD15 and CD30 and negative for CD45, consistent with classical Hodgkin Lymphoma, nodular sclerosis, grade 2. Imaging also revealed nonbulky disease in the anterior mediastinum, and no bone marrow biopsy was performed (Figure 1(a)). The patient declined conventional therapy, instead seeking 5 months of alternative treatments without response. Her disease remained stable, and thus she continued to decline therapy. 

Five years later, she developed pruritus, night sweats, and dysphagia. One year later, she developed progressively severe dysphagia with a 12 kg weight loss, significant dyspnea, productive cough, and was admitted to the hospital to receive intravenous antibiotics for pneumonia. Imaging revealed significant adenopathy in the neck, mediastinum, and abdomen, groin as well as hepatosplenomegaly (Figure 1(a)). In addition, she had partial compression of the superior vena cava and trachea by the mediastinal mass, cavitary lesion in the upper lobe of the right lung, and evidence of tracheoesophageal and tracheomediastinal fistulae (arrow in lower Figure 1(a)). Excisional biopsy of a supraclavicular lymph node showed sheets of Reed-Sternberg cells with focal necrosis and dense but limited fibrosis. The large neoplastic cells were positive for CD15 and CD30 but negative for CD20 and CD45, consistent with classical Hodgkin lymphoma, nodular sclerosis subtype. Bone marrow examination was without disease. A bronchoscopy and barium swallow (Figure 2) identified fistulous tracts connecting the trachea with the esophagus and the mediastinum. A gastrostomy tube and tracheal stent (covered and self-expanding, Microinvasive, Boston Scientific, Natick, MA) were placed to minimize the risk of recurrent aspiration pneumonia.

Despite requiring supplemental oxygen and weighing only 39 kilograms, therapy was initiated 73 months after her initial diagnosis with doxorubicin, vinblastine, and dacarbazine (AVD). Bleomycin was not given due to lung disease and oxygen requirement. She had minimal therapy-related toxicities which responded well to supportive care. After 2 cycles of therapy, restaging studies showed a 75% decrease of her mediastinal mass, but the fistulae remained unchanged. Her cough significantly improved and oxygen requirement normalized. She continued on therapy, completing 8 cycles and gaining over 5 kilograms. Restaging CT scans demonstrated a near complete resolution of her mediastinal disease and fistulae, and no other disease was noted. A posttreatment PET/CT demonstrated a non-FDG avid residual mediastinal mass. At restaging imaging 3 months later, the mass persisted unchanged and the patient declined biopsy or further intervention.

 Approximately two years after completing therapy, she remained without disease but had frequent upper respiratory infections. Her stent was exchanged, and eventually removed. After removal, her frequent infections disappeared. She remains without evidence of disease six years removed from initiation of therapy and eleven years from diagnosis.

## 3. Discussion

Due to successful therapies, the natural history of untreated Hodgkin lymphoma is extremely difficult to ascertain [3, 4]. Prior to establishing the potentially curative role of chemotherapy, surgical resection and radiation therapy were the primary initial therapies [5]. Systemic disease was often underappreciated due to available technology, and thus patients treated for local disease often relapsed in distant, untreated sites. Estimates of survival from this era do not reflect the truly untreated state, but allow general estimates. In a series of patients treated with radiation +/− surgery, 5 year overall survival was 26.9% [6].

Aside from rare case reports, only two large series of untreated patients exist [7]. Craft [8] and Greco et al. [9] evaluated 52 and 80 biopsy-proven cases, respectively, of untreated Hodgkin lymphoma from 1910–1962. The median overall survival from Craft's series was 16.6 months, with a 3 year survival of 15.4%, and greater than 5 year survival of less than 6%. The median overall survival from Greco's series was 19.7 months, with 10% surviving 5 years. Historical series lack complete information to allow assessment of modern risk prognostic scoring systems. Furthermore, they may underestimate survival, due to relatively delayed diagnosis from lack of access to medical care or requirement of clinically apparent disease. Regardless of these caveats, our patient did quite well with over six years between diagnosis and morbid illness.

Malignant tracheoesophageal fistulae are relatively rare, are most commonly seen with esophageal or lung cancer, and are associated with high mortality [10, 11]. Tracheal fistulas are a rare complication of Hodgkin lymphoma and typically have a much better prognosis than fistulae associated with other malignancies. A large retrospective review identified a total of 212 patients with respiratory-esophageal fistula at a major center from 1926–1988 [11]. Of these patients, only 2 had Hodgkin lymphoma. In general, the diagnosis of a respiratory-esophageal fistula is an extremely morbid disease, with a median overall survival in this series of only 35 days with sepsis/pneumonia as the leading cause of death. 

A literature review of 22 reported patients with Hodgkin lymphoma and tracheaesophageal fistula [12] showed that the fistula may occur with any of the classical Hodgkin lymphoma subtypes and is usually associated with active disease at the site of fistula. However, trachea-esophageal fistula may also develop during radiotherapy or chemotherapy from rapid tumor necrosis or as a late complication after radiotherapy due to erosion of scar tissue. Rarely, tracheoesophageal fistulae can be the initial sign of late relapsing disease [13]. The majority of fistulae occurred in the upper two-thirds of the esophagus, and 67% treated solely with chemotherapy and/or radiotherapy closed without surgical intervention [12, 14]. A review of all lymphomas involving the esophagus seen over 45 years at Mayo Clinic identified 27 cases with 6 developing a fistula [15]. Our case is unique from these previous reports of Hodgkin lymphoma with tracheoesophageal fistula as our patient developed her complications during a five-year period where she refused therapy.

The most important feature of management of tracheoesophageal fistula is early identification and initiation of therapy [16]. The recommended initial therapy is placement of a stent, either in the trachea or esophagus [16]. Stenting prevents further soilage of the respiratory tract and thus decreases the risk of pneumonia or respiratory compromise to allow for malignancy specific therapy. In addition, feeding via a gastrostomy tube further reduces the risk of pulmonary soilage, aspiration pneumonia, and mediastinitis. Commonly used stents, such as the covered self-expanding stent used in our patient, have been successfully removed without complication years after initial placement without complication [17]. 

In conclusion, we present the case of a patient with two extremely rare conditions, untreated Hodgkin lymphoma, and tracheoesophageal fistula. Despite six years lapsing from diagnosis to initiation of therapy, the patient had an excellent response to standard chemotherapy. The typically morbid tracheoesophageal fistula was managed successfully with early placement of both a tracheal stent and gastrostomy tube. The fistula closed with chemotherapy alone, consistent with other follow-up studies suggesting that long-term closure of the tracheoesophageal fistula is possible with conservative treatment in patients with Hodgkin lymphoma. As therapy for Hodgkin lymphoma often achieves excellent outcomes, we feel that even patients with a moribund presentation should be considered for aggressive therapy with curative intent.

## Figures and Tables

**Figure 2:**